# Supplementary material for: COVID-19 and the kidney: A retrospective analysis of 37 critically ill patients using machine learning
Source: PLoS One. 2021 May 20;16(5):e0251932. doi: 10.1371/journal.pone.0251932 (PMC8136725; doi:10.1371/journal.pone.0251932)
Supplement: Attachment — Submitted filename: Response to Reviewers Covid ML.doc [file pone.0251932.s001.doc]

Medizinische Klinik I · Nephrologie · Oberdürrbacher Str. 6 · 97080 Würzburg

**SCHWERPUNKT**

**NEPHROLOGIE**

Leiter: Prof. Dr. C. Wanner

Tel.: (09 31) 201-39301

(09 31) 201-39030

Fax: (09 31) 201-639300

Wanner_C@medizin.uni-wuerzburg.de

Station M32:

Arzt: Tel.: (0931) 201-43210

Pflege: Tel.: (0931) 201-43200

Nierentransplantation:

Tel.: (0931) 201-43214

Mobil: (0931) 201-43914

Hämodialyse:

Tel.: (09 31) 201-43700

IPD/CAPD:

Tel.: (0931) 201-43700

Nieren- & Hochdruckambulanz:

Tel.: (0931) 201-39432; -39126

Transplantationsbüro:

Tel.: (09 31) 201-39305

Nephrologisches Labor:

Tel.: (09 31) 201-39727

Fabry Ambulanz:

Tel.: (0931) 201-39126

Tel.: (09 31) 2 01-57057 (Pforte)

Tel.: (09 31) 2 01-0 (Zentrale)

www.medizin.uni-wuerzburg.de

| Ihre Nachricht vom: | Bitte bei Antwort angeben: | Telefon: (09 31) 2 01 – 39030; -39301 | | Würzburg, 30. November 2026 |
| --- | --- | --- | --- | --- |
| Ihr Zeichen: | Unser Zeichen: | Telefax: (09 31) 2 01 – 639300 | |  |

Dear Editors,

Please find attached our revised manuscript with the title “COVID-19 and the kidney: A retrospective analysis of 37 critically ill patients using machine learning”

We are very grateful for the suggestions and criticisms; we have now addressed the comments and suggestions provided by the reviewer, which in our opinion add considerably to the quality of our work. We revised the manuscript, added contemporary literature and edited the material/methods part.

We hope that the revised manuscript will find your approval for publication in *PlosOne*. Again, we thank you very much for your attention and interest in our work and look forward to hear from you.

Sincerely,

Anna Laura Herzog on behalf of all Authors

**Point-by-Point Reply**

With regard to the individual suggested changes, we would politely like to draw your attention to the following items:

We have indicated that raw data from this study are available upon request, knowing that this is not PlosOne's policy. In our study the need for informed consent from individual patients was waived by the ethics committee of the university of Wuerzburg. Data contain potentially identifying and sensitive patient information. According to local requirements, anonymized individual patient level data can only be shared upon request and prior written permission from the Ethics Committee of the

University of Wuerzburg (Versbacher Str. 9, 97078 Wuerzburg, Germany, ethikkommission@uniwuerzburg.de) and the data protection officer of the University hospital of Wuerzburg. All other data are provided in the manuscript. The entire R-Package can be downloaded under <https://github.com/vonjd/OneR>, the link is of course provided.

**Reply to reviewer**

We thank the reviewer for the critical evaluation of our work. We are glad that our manuscript was well received (“*technically sound, data support the conclusions, very important research question*”) and would therefore like to reply to the issues raised by drawing the attention to following items:

Is the manuscript presented in an intelligible fashion and written in standard English? “No”

We apologize that our manuscript does not meet the required language standards. The manuscript was under professional editing, we will ask for a revision from the responsible editor once again

Ad 1.: Sample size is small, reducing the generalizability of study results: please mention this point among the limitations of the manuscript

Thank you for this valuable suggestion. We are absolutely aware that our case counts are too small for a valid statement. Our intention was to provide an overview of the methodology, its application and sources of error. Regarding the predictive power of proteinuria, our results are in line with previous reports despite the small number of patients, although even in much larger studies the exact correlation is not clear. To explain different ML methods, the small sample size was also sufficient, demonstrating sources of error such as overfitting in a simplified way. We have mentioned this again in the discussion.

Ad 2. “Curiously, I saw that Authors reported in the results a copy of the syntax used to run the OneR algorithm. This is unusual in the context of an original research. I suggest to delete that part”

Thank you for this comment. We have tried to explain ML methods in a simple way. The OneR package is an important tool for our approach. We have shown the part of the syntax to give an unfamiliar user an insight into the quite intuitive output of the program. We realize that this is unusual and have corrected that.

Ad 3.: “Within the machine learning techniques, there are interesting approach which are useful in the case of small sample size and a large amount of data. These include the penalized regression such as the Lasso regression or the Ridge regression. Did you consider using some of them?”

Thank you for this important remark. The paper is intentionally focused on machine learning methods, which closes a gap in the current literature. We considered regularization methods for linear regression (e.g. Lasso and Ridge regression), however, this more traditional approach would have gone beyond the scope of this work. A second more technical reason is that the target variable of Lasso and Ridge Regression is numerical whereas our target variables are categorical. Although Lasso and Ridge regression models can be interpreted to some degree they also suffer from some issues. Our approaches (i.e. OneR and decision trees) can be understood very easily and have a clear medical interpretation as we tried to show in the paper.

Ad 4.: “The discussion section could be implemented by generating more hypotheses on the link between kidney measured and cardio-renal risk. To this aim, please read and if possible cite the manuscript “doi: 10.3390/jcm9082506” and “doi: 10.2217/bmm-2020-0201”.

Thank you for this very interesting approach. In fact, we have recorded several cardiac parameters. We could also show that severe AKI in combination with cardiac involvement increases the risk of death. A very interesting approach for our future investigation would be to see if potential cardiac deterioration due to COVID-19 can also be detected early this way, we will follow up on this.

We have largely implemented your valuable suggestions on methods and discussion, added more contemporary literature, structured the main text to make citations more clearly and explained or removed individual sentences. We have changed the manuscript and thank the reviewer for pointing out these important issues.

Kind regards,

Anna Laura Herzog

On behalf of the authors
